# Selective pruning in pineapple plants as means to reduce heterogeneity in fruit quality

**DOI:** 10.1186/s40064-015-0907-9

**Published:** 2015-03-14

**Authors:** V Nicodème Fassinou Hotegni, Willemien J M Lommen, Euloge K Agbossou, Paul C Struik

**Affiliations:** Centre for Crop Systems Analysis, Wageningen University, Droevendaalsesteeg 1, 6708 PB Wageningen, The Netherlands; Faculté des Sciences Agronomiques, Université d’Abomey Calavi, 01 BP 526 Cotonou, Benin

**Keywords:** Ananas comosus, Cv. Pérola, Pruning time, Slips, Thinning, Uniformity, Variation in quality, Variation within a field

## Abstract

Heterogeneity in fruit quality (size and taste) is a major problem in pineapple production chains. The possibilities were investigated of reducing the heterogeneity in pineapple in the field by pruning slips on selected plants, in order to promote the fruit growth on these plants. Slips are side shoots that develop just below the pineapple fruit during fruit development. Two on-farm experiments were carried out in commercial fields in Benin with a cultivar locally known as Sugarloaf, to determine (a) the effect of slip pruning on fruit quality; (b) whether the effect of slip pruning depends on the pruning time; and (c) whether slip pruning from the plants with the smallest infructescences results in more uniformity in fruit quality. A split-plot design was used with pruning time (2 or 3 months after inflorescence emergence) as main factor and fraction of pruned plants (no plants pruned (control); pruning on the one-third plants with the smallest infructescences; pruning on the two-thirds plants with the smallest infructescences; pruning on all plants) as sub-factor. Fruit quality characteristics measured at harvest were the fruit (infructescence + crown) weight and length, the infructescence weight and length, the crown weight and length, the ratio crown length: infructescence length, the total soluble solids, the juice pH and the flesh translucency. Results indicated that pruning of slips of any fraction of the plants at 2 or 3 months after inflorescence emergence did not lead to a consistent improvement in quality or uniformity. Consequently it is not recommended to farmers in Benin to prune the slips.

## Introduction

In developing countries, many producers –especially the smallholder producers– face difficulties in entering the international market because of the high quality standards and the need to supply high and regular quantities of product (Murphy 2012). Nowadays, the uniformity in product quality also has become an important criterion. As a proof of that, the Codex Alimentarius, an organization focusing on the establishment of food quality and safety rules for export products to which most developing countries belong, elaborated a set of export criteria for individual food quality attributes as well as for acceptable product heterogeneity (Codex Alimentarius 2005). A recent study on pineapple [*Ananas comosus* (L.) Merrill] supply chains in Benin revealed that heterogeneity in quality attributes such as fruit weight, taste, firmness and flesh translucency was a constraint to the success of the chain (Fassinou Hotegni et al. 2014a). Heterogeneity in quality is caused by many factors including the environmental conditions and cultural practices underlying its production (Luning and Marcelis 2006). It then becomes important to find ways to reduce heterogeneity in fruit quality by designing crop management strategies yielding a more uniform product quality at harvest.

The most important pineapple cultivar in Benin is the sweet cultivar locally known as Sugarloaf –but possibly equal to cv. Pérola– grown by 97% of the pineapple growers (Fassinou Hotegni et al. 2014a). In this cultivar type and several other types like cv. Singapore Spanish grown in, e.g., South Asia, three development phases exist: the vegetative phase (from planting to flowering induction); the generative phase (from flower initiation to fruit maturity), and the propagative phase in which new shoots are produced (begins at the generative phase and continues after the fruit has been harvested) and which partly overlaps with the generative phase. Cultural practices to control heterogeneity are carried out mainly before flowering induction. Heterogeneity in pineapple fruit quality (fruit weight and length attributes) at harvest is clearly associated with heterogeneity in plant vigor at flowering induction time (Fassinou Hotegni et al. 2014b), and consequently cultural practices achieving uniform plant development before flower induction (like planting material grading by size at planting time) have received considerable attention (Bartholomew et al. 2003, Py et al. 1987). Yet smallholder systems still show a high heterogeneity in quality (Fassinou Hotegni et al. 2015). The present research focused on the potential of practices applied after flowering induction to reduce the heterogeneity in pineapple fruit quality, i.e., in total fruit (infructescence + crown) weight and length, infructescence weight and length, crown weight and length, the ratio crown length: infructescence length, the total soluble solids (TSS) in the pineapple juice, the juice pH, and the flesh translucency.

Due to the overlap between the generative phase and the propagative phase the generative phase is not only characterized by development and growth of the fruit; also new shoots develop during that phase, such as slips (produced on the peduncle at the base of the fruit), hapas (produced above ground from the stem at the junction of the stem and the peduncle), suckers (side shoots originating on the stem) (Hepton 2003) and the crown. These vegetative organs can be used as propagules for planting a next crop. The most common shoots produced in the Pérola pineapple type and cv. Singapore Spanish are the slips and the crown. The slips are initiated just after the end of the initiation of the florets (Kerns et al. 1936). Studies on the effect of removing slips –called pruning or thinning– on the fruit size gave contradictory results. Wee and Ng (1970) removed all slips in cv. Singapore Spanish in excess to two slips that were kept on the plants and found no significant effect of slip pruning on fruit weight and fruit length. Similar results were also found by De Lima et al. (2001) on cv. Pérola. Norman (1976) removed the slips in Sugarloaf pineapple when the fruits started to develop and found that slip pruning increased fruit weight but had no effect on TSS concentration in the fruit juice. Recent glasshouse studies on cv. Smooth Cayenne suggested that slips could be an important source of assimilates for fruit growth and maintenance (Marler 2011) which again suggests that slip removal may affect fruit quality. Such inconsistent results emphasize the need to improve the understanding of the effect of slip pruning on fruit quality.

Since the production of the slips overlaps with fruit development and growth, slips may compete with the fruit for assimilates available in the plant especially at an earlier stage of their development when they are not yet capable of producing their own assimilates. Thus, earlier slip pruning may have more positive effects on average fruit quality than later pruning. It was shown in pineapple that the least developed plants at flower induction produce lighter fruit than well-developed plants (Fassinou Hotegni et al. 2014b). We therefore assume that a higher uniformity in fruit weight and length might be achieved by early pruning of the slips of the least developed plants. A practical criterion for farmers to identify the least developed plants after flower induction would be the length of the developing infructescence. The objectives of this paper are to determine (1) the effect of slip pruning on the fruit quality, namely fruit (infructescence + crown) weight and length, infructescence weight and length, crown weight and length, the ratio crown length: infructescence length, the TSS in the pineapple juice, the juice pH and the flesh translucency; (2) whether the effect of slip pruning depends on the pruning time; and (3) if slip pruning from the plants with the smallest infructescences results in more uniformity in fruit quality.

## Materials and methods

### Experimental sites and set up

**Figure 1:**
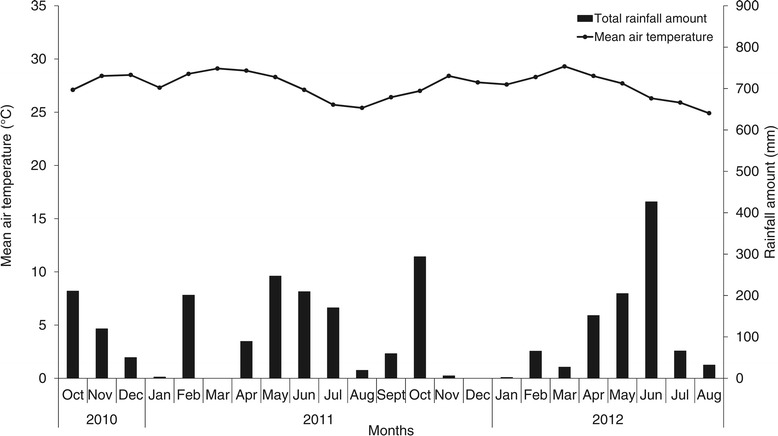
**Variation in mean air temperature and monthly rainfall during the experimentation period (October 2010 to August 2012).**

**Figure 2:**
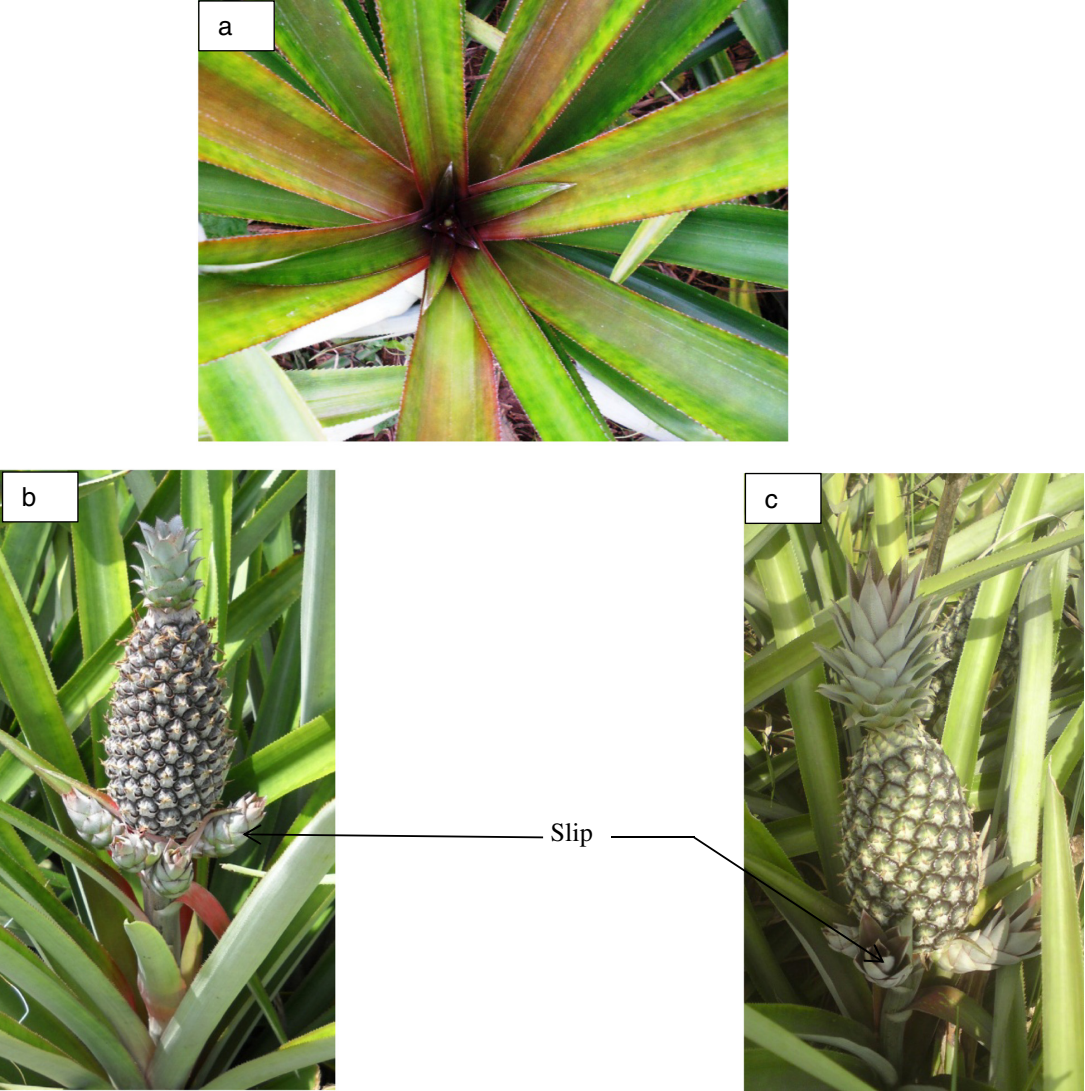
**Pineapple plants at different stages of the generative phase: (a) flower emergence at the center of the leaf rosette; (b) pineapple plant at 2 MIE (months after inflorescence emergence) showing the slips; (c) pineapple plant at 3 MIE showing the slips.** Pictures (**a**), (**b**) and (**c**) were taken from different plants.

Two on-farm experiments (Expt 1 and Expt 2) were conducted in two commercial pineapple farms (Farm A and Farm B) in the Atlantic department in the south of Benin between October 2010 and August 2012. Different producers of a cultivar locally known as Sugarloaf, but possibly equal to cv. Pérola, were selected per experiment based on (a) the age of their pineapple crop being close to the common artificial flowering induction time and (b) whether they applied the common practices described by Fassinou Hotegni et al. (2012) for this cultivar, as suggested by Mutsaers et al. (1997) for on-farm studies. Cultivar Sugarloaf was selected because (1) it is grown by 97% of the pineapple producers in the department (Fassinou Hotegni et al. 2014a) and (2) it produces numerous slips during the generative phase (Fassinou Hotegni et al. 2014b, Norman 1976). Information on the farms and cultural practices from planting until harvest time is presented in Table 1; mean monthly temperature and total monthly rainfall amount during the experimentation period are depicted in Figure 1. In each experiment, a split-plot design was used with slip pruning time [2 and 3 months after inflorescence emergence (MIE); Figure 2] as the main factor and fraction of plants per plot selected for pruning of slips (none, one-third, two-thirds, all) as the split factor. The pruning time 2 MIE was selected because at that time the slips and fruit were developed well enough to allow pruning without damaging the growing fruit (Figure 2B). The pruning time 3 MIE was selected because at that time the slips were well-developed when compared with their size at 2 MIE (Figure 2C). Plants for slip pruning (i.e., fraction of plants pruned) were the one- or two-third(s) plants (per plot) with the smallest infructescences (in length) at the time of pruning. The infructescence length was selected because it is a practical and easy criterion for farmers to identify the least developed plants and because a pre-experiment (not reported here) had shown that the infructescence length at 2 MIE was correlated with fruit weight and infructescence length at harvest (*r* = 0.63 and 0.64 respectively). Each experiment had four replicated blocks. Each net plot consisted of 60 plants arranged in 6 lines of 10 plants each; the number of plants selected for pruning was fixed at 0, 20, 40 or all 60 per net plot. Each net plot was surrounded by at least 2 guard rows and 2 guard plants in a row. The pineapple fruits were harvested following farmers’ practice which was at the moment when the skin color had started to change from green to yellow in at least 25% of the plants in a net plot (i.e., 15 out of 60 plants). All fruits per plot were harvested on that day and were individually processed.

**Table 1:** **Information on sites and cultural practices for the two experiments with cv. Sugarloaf**

**Field information and cultural practices**	**Expt 1**	**Expt 2**
Location	06°36′35.7“N and 02°14′28.7”E	06°35′06.4“N and 02°15′55.4”E
Municipality (district)	Zè (Tangbo Djevie)	Zè (Tangbo Djevie)
Soil type	Ferralitic soil	Ferralitic soil
Climate	Subequatorial	Subequatorial
Planting time^a^	October 2010	March 2011
Type of planting material used^a^	Slips	Slips
Planting material treatment before planting^a^	No treatment	No treatment
Plant arrangement at planting	Flat beds of two rows	Flat beds of two rows
Plant spacing (cm): BP^b^ × BR^c^/BDR^d^	35 × 47/75	40 × 50/70
Plant density (plants/m^2^)	4.68	4.17
First Urea (46 N) + NPK (10-20-20) application	6 MAP^e^ (18 April 2011)	7 MAP (11 October 2011)
*Application form*	Solid at the base of the plants	Solid at the base of the plants
*Dose per plant (g Urea + g NPK)*	6 + 3	6 + 4
Second Urea (46 N) + NPK (10-20-20) application	12 MAP (13 October 2011)	11 MAP (16 February 2012)
*Application form*	Solid at the base of the plants	Solid at the base of the plants
*Dose per plant* (*g Urea* + *g NPK*)	3 + 7	3 + 7
Artificial flowering induction time	13 MAP (13 November 2011)	12 MAP (17 March 2012)
Inflorescence emergence	14 MAP (17 December 2011)	13 MAP (20 April 2012)
First removal of slips (2 MIE^f^)	16 MAP (17 February 2012)	15 MAP (20 June 2012)
Second removal of slips (3 MIE)	17 MAP (17 March 2012)	16 MAP (20 July 2012)
Weed control	Hand weeding	Hand weeding
Harvest time	18 MAP (15, 16, 17 and 18 April 2012)	17 MAP (20, 21, 22 and 23 August 2012)

^a^: Information gathered from pineapple producer (field owner); ^b^: Between plants; ^c^: Row width; ^d^: Between double rows; ^e^: Months after planting; ^f^: Months after inflorescence emergence.

### Collected data

Data were collected on all individual plants per net plot before pruning and at harvest. Before pruning, the infructescence length and the number of slips per plant were recorded. At harvest time, only data on fruit quality attributes were collected. Fruit quality attributes included some listed in the Codex Alimentarius such as the ratio crown length: infructescence length, the TSS in the pineapple juice and the flesh translucency, and some such as fruit and infructescence weight (mentioned in the Codex as size attributes) and juice pH (affecting the taste of the fruit) of high importance for pineapple consumers in some countries such as Benin, Nigeria, Burkina Faso and Niger (Fassinou Hotegni et al. 2014a), and some such as fruit, crown and/or infructescence lengths and weights underlying the fruit weight, the ratio crown length: infructescence length, or needed for their assessment. Data on fruit quality collection followed the procedures described by Fassinou Hotegni et al. (2014b), with TSS being measured in the pineapple juice in °Brix using a hand refractometer and the juice pH using a hand-held pH meter. Flesh translucency was based on the percentage of fruit flesh that was translucent; it was visually estimated on a cut half pineapple following the method of Paull and Reyes (1996).

### Data analysis

Data were analyzed using GenStat for Windows 15th Edition (VSN International 2012).

The initial status of the plants at pruning time was described in two ways. First, the proportion of plants with slips and the total number of slips produced per plant were calculated per plot and checked for being similar across treatments. A two-way ANOVA for a split-plot design was used; the proportion of plants with slips was transformed using arcsine transformation on the square root of the proportions before the analysis. Second, sextiles were calculated per plot. Plants were ranked according to infructescence length from the smallest to the highest values per plot and then allocated to six classes. The number of plants with slips was counted per class. Plants (n = 960) from all treatments at one pruning time were combined per sextile and graphs were plotted to evaluate how the proportion of plants without slips and the number of slips per plant in plants with slips varied in the sextiles at each pruning time.

Because not all plants had produced slips, two data sets were created for evaluating fruit quality attributes: (1) *a data set based on all plants per plot* (with or without slips at pruning time) and (2) *a data set based on plants with slips* at pruning time. A two-way ANOVA for a split plot design was performed on each data set to test the effect of pruning time and fraction of plants pruned on the average quality of the fruit quality attributes and on fruit quality heterogeneity. Flesh translucency data were transformed using square root transformation $\left(\sqrt{x+0.5}\right)$ before analysis (Bartlett 1936, Gonzalez 2009). Fruit quality heterogeneity was calculated per plot using the coefficient of variation (CV), i.e., the measure of the variability in the value in a population relative to the mean, for the two data sets: all plants and plants with slips at pruning time. When the F value was significant, LSD was used to separate means of average quality or CV in quality.

## Results

### Initial status of the plants at pruning time

**Figure 3:**
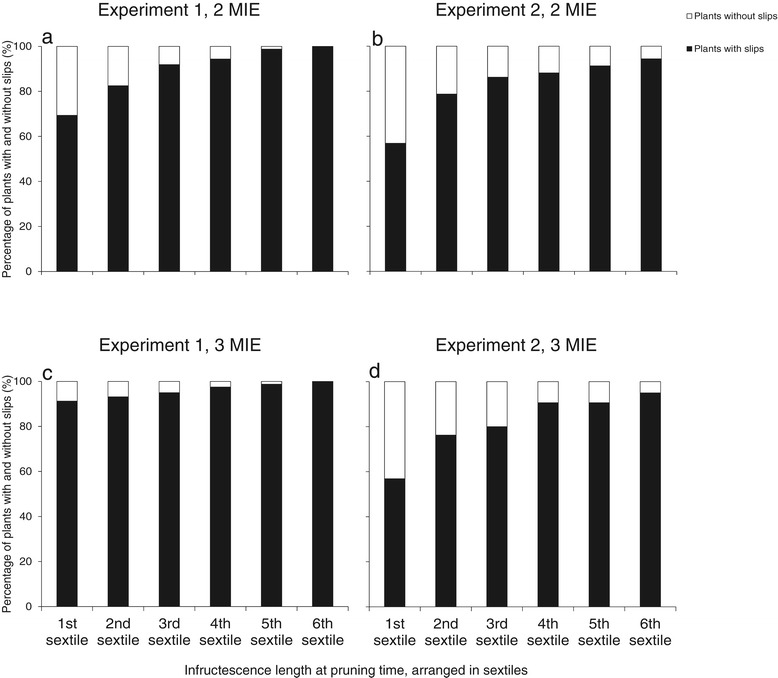
**Proportion of plants with and without slips as function of infructescence length arranged from smallest (1st sextile) to highest (6th sextile) at 2 MIE (months inflorescence emergence) (a and b) and 3 MIE (c and d) in Experiments 1 and 2.** Each diagram is based on 960 plants.

The pruning time, the fraction of plants pruned and their interaction were confirmed to have no effect on the proportion of plants with slips and the number of slips at pruning (Table 2). This shows that plants with and without slips were evenly distributed across the plots at the moment the treatments started. In Expt 1, there were fewer plants without slips than in Expt 2 (Figure 3); in addition, the total number of slips produced in Expt 1 was higher than that produced in Expt 2.

**Table 2:** ***P***-**values of the F ratios testing the effect of pruning time, fraction of plants pruned and their interaction on the proportion of plants with slips and the total number of slips produced**

	**Expt 1**	**Expt 2**
Proportion of plants with slips		
Pruning time (PT)	0.269	0.860
Fraction plants pruned (FP)	0.101	0.747
PT × FP	0.307	0.419
Total number of slips		
Pruning time (PT)	0.738	0.762
Fraction plants pruned (FP)	0.789	0.696
PT × FP	0.312	0.378

**Figure 4:**
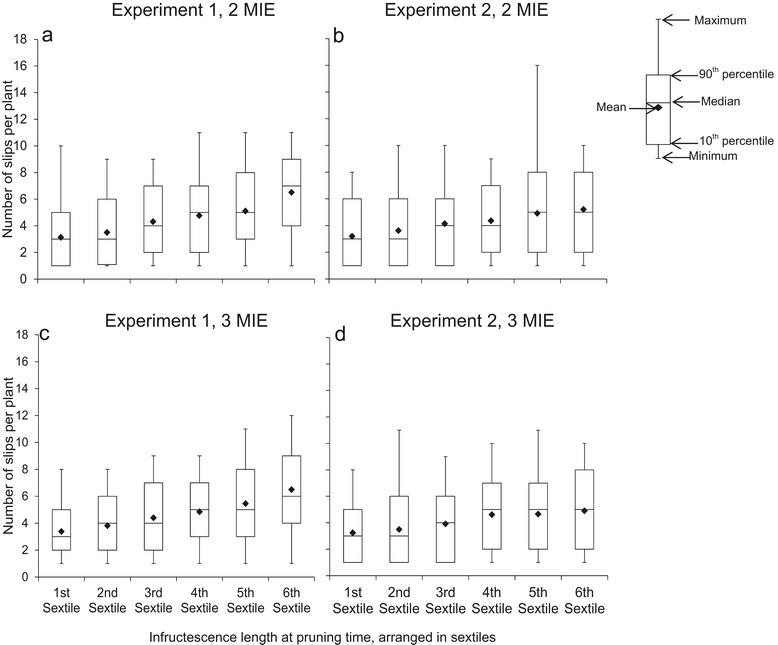
**Boxplots with whiskers showing the mean, median, minimum, maximum, and the 10th and 90th percentiles of number of slips per plant in plants with slips within each infructescence length category arranged from smallest (1st sextile) to highest (6th sextile) at 2 MIE (months after inflorescence emergence) (a and b) and 3 MIE (c and d) in Experiments 1 and 2.**

Infructescence length at pruning ranged from 5.5-20 cm in Expt 1 and from 6.6-19.0 cm in Expt. 2 when pruning was carried out at 2 MIE; at 3 MIE, infructescence length ranged from 8.5-24.5 cm in Expt 1 and 8.5-21.5 cm in Expt 2 (data not shown). When plants per plot were arranged into sextiles based on their infructescence length at pruning time, plants in lower sextiles –in which fractions most of the plants that had to be pruned fell– less likely had produced slips (Figure 3) – and if so, the number of slips was lower (Figure 4). This meant that a possible effect of pruning on fruit quality was diluted by the plants that could not be pruned because they did not have slips. Therefore, data were split into two sets: (1) *a data set based on all plants per plot* (with or without slips at pruning time) and (2) *a data set based on the plants with slips* at pruning time. Infructescence length at pruning in the latter data set ranged from 6.0-20 cm in Expt 1 and 8.0-19.0 cm in Expt 2 when pruned at 2 MIE, and from 8.5-24.5 cm in Expt 1 and from 8.5-21.5 cm in Expt 2 when plants were pruned at 3 MIE.

The *data set based on all plants per plot* is useful for showing the relevance of pruning for commercial practice and the *data set based on the plants with slips* for understanding the effect of slip pruning *per se*.

### Effects of fraction of plants pruned and pruning time on fruit quality

In both data sets –data on all plants per plot and data based on the plants with slips at pruning time– the interaction between pruning time and fraction of plants pruned was not significant for any of the quality attributes and main effects were only incidentally significant (Table 3). In both data sets, the fraction of plants pruned had no significant effect on average quality, except on juice pH in Expt 1 (Table 3), where pruning of the two-thirds plants with the smallest infructescences led to higher juice pH than no pruning or pruning all plants (Table 4). This trend in juice pH was not found in Expt 2.

**Table 3:** ***P***-**values of the F ratios from ANOVA for the effects of pruning time, fraction of plants pruned and their interaction on average pineapple fruit quality attributes and variation in quality (CV) in two experiments for data based on all plants and on plants with slips only**

**Fruit quality/Factor**	**Effect on average fruit quality**				**Effect on variation in fruit quality**			
	**All plants**		**Plants with slips**		**All plants**		**Plants with slips**	
	**Expt 1**	**Expt 2**	**Expt 1**	**Expt 2**	**Expt 1**	**Expt 2**	**Expt 1**	**Expt 2**
Fruit weight (kg)								
Pruning time (PT)	0.923	0.754	0.995	0.740	0.048 *	0.392	0.194	0.183
Fraction plants pruned (FP)	0.974	0.363	0.953	0.286	0.917	0.758	0.693	0.342
PT × FP	0.515	0.287	0.668	0.132	0.388	0.570	0.717	0.032 *
Infructescence weight (kg)								
Pruning time (PT)	0.892	0.791	0.968	0.776	0.106	0.358	0.170	0.152
Fraction plants pruned (FP)	0.985	0.465	0.959	0.395	0.886	0.851	0.606	0.490
PT × FP	0.507	0.281	0.661	0.120	0.335	0.347	0.678	0.028 *
Crown weight (kg)								
Pruning time (PT)	0.021 *	0.528	0.002 **	0.553	0.058	0.691	0.141	0.954
Fraction plants pruned (FP)	0.158	0.510	0.178	0.657	0.120	0.735	0.111	0.699
PT × FP	0.395	0.686	0.434	0.845	0.448	0.790	0.666	0.950
Fruit length (cm)								
Pruning time (PT)	0.923	0.890	0.954	0.886	0.070	0.930	0.016 *	0.891
Fraction plants pruned (FP)	0.995	0.404	0.986	0.520	0.295	0.699	0.755	0.772
PT × FP	0.961	0.356	0.966	0.495	0.995	0.247	0.841	0.672
Infructescence length (cm)								
Pruning time (PT)	0.744	0.796	0.819	0.783	0.344	0.573	0.972	0.909
Fraction plants pruned (FP)	0.973	0.557	0.915	0.478	0.425	0.811	0.903	0.767
PT × FP	0.906	0.524	0.972	0.370	0.683	0.311	0.737	0.360
Crown length (cm)								
Pruning time (PT)	0.297	0.551	0.177	0.613	0.353	0.795	0.655	0.559
Fraction plants pruned (FP)	0.897	0.609	0.893	0.687	0.041 *	0.765	0.134	0.678
PT × FP	0.716	0.713	0.697	0.846	0.297	0.247	0.200	0.505
Ratio crown length: infructescence length								
Pruning time (PT)	0.543	0.422	0.587	0.404	0.865	0.898	0.910	0.489
Fraction plants pruned (FP)	0.830	0.681	0.750	0.645	0.337	0.671	0.606	0.572
PT × FP	0.754	0.754	0.858	0.678	0.294	0.064	0.241	0.130
Total soluble solids (°Brix)								
Pruning time (PT)	0.914	0.868	0.901	0.914	0.700	0.353	0.858	0.418
Fraction plants pruned (FP)	0.531	0.332	0.587	0.302	0.973	0.143	0.816	0.076
PT × FP	1.000	0.416	0.998	0.477	0.966	0.498	0.871	0.589
Juice pH								
Pruning time (PT)	0.838	0.810	0.691	0.796	0.606	0.359	0.706	0.312
Fraction plants pruned (FP)	0.011 *	0.781	0.013 *	0.742	0.775	0.273	0.703	0.347
PT × FP	0.339	0.397	0.291	0.447	0.806	0.776	0.848	0.775
Flesh translucency								
Pruning time (PT)	0.911	0.947	0.817	0.967	0.871	0.987	0.994	0.970
Fraction plants pruned (FP)	0.722	0.324	0.842	0.283	0.903	0.935	0.807	0.778
PT × FP	0.072	0.140	0.113	0.274	0.151	0.142	0.184	0.163

Values in *bold* indicate significant *P*-values, *P* < 0.05.

*Significant at the 0.05 probability level; **Significant at the 0.01 probability level.

**Table 4:** **Average and variation in different fruit quality attributes in two experiments, for data based on all plants and plants with slips only**

	**Average fruit quality**				**Coefficient of variation in fruit quality**			
	**All plants**		**Plants with slips**		**All plants**		**Plants with slips**	
	**Expt 1**	**Expt 2**	**Expt 1**	**Expt 2**	**Expt 1**	**Expt 2**	**Expt 1**	**Expt 2**
Fruit weight (kg)	0.96	0.91	0.97	0.96	0.26	0.25	0.25	0.23
*Pruning time*								
*2MIE* ^*a*^	*0.95*	*0.93*	*0.97*	*0.97*	***0.27 b*** ^***h***^	*0.26*	*0.25*	*0.23*
*3MIE* ^*b*^	*0.96*	*0.90*	*0.97*	*0.94*	***0.25 a***	*0.25*	*0.24*	*0.22*
*Pruning treatments at 2MIE*								
*NP* ^*c*^	*0.95*	*0.96*	*0.97*	*1.01*	*0.26*	*0.25*	*0.25*	***0.22 b***
*1/3P* ^*d*^	*0.97*	*0.85*	*0.99*	*0.91*	*0.26*	*0.27*	*0.25*	***0.22 b***
*2/3P* ^*e*^	*0.96*	*0.93*	*0.98*	*0.96*	*0.27*	*0.26*	*0.26*	***0.23 b***
*AP* ^*f*^	*0.91*	*0.98*	*0.96*	*1.01*	*0.28*	*0.26*	*0.26*	***0.24 b***
*Pruning treatments at 3MIE*								
*NP*	*0.95*	*0.85*	*0.96*	*0.88*	*0.24*	*0.26*	*0.23*	***0.24 b***
*1/3P*	*0.96*	*0.90*	*0.97*	*0.94*	*0.26*	*0.24*	*0.25*	***0.22 b***
*2/3P*	*0.94*	*0.93*	*0.95*	*0.98*	*0.25*	*0.23*	*0.25*	***0.19 a***
*AP*	*1.01*	*0.92*	*1.02*	*0.96*	*0.24*	*0.26*	*0.23*	***0.22 b***
Infructescence weight (kg)	0.86	0.77	0.88	0.81	0.29	0.30	0.27	0.26
*Pruning treatments at 2MIE*								
*NP*	*0.85*	*0.81*	*0.88*	*0.86*	*0.29*	*0.30*	*0.28*	***0.26 ab***
*1/3P*	*0.87*	*0.71*	*0.88*	*0.76*	*0.29*	*0.31*	*0.27*	***0.25 ab***
*2/3P*	*0.86*	*0.77*	*0.88*	*0.80*	*0.29*	*0.31*	*0.29*	***0.28 b***
*AP*	*0.81*	*0.83*	*0.85*	*0.86*	*0.31*	*0.29*	*0.28*	***0.27 b***
*Pruning treatments at 3MIE*								
*NP*	*0.85*	*0.71*	*0.87*	*0.74*	*0.26*	*0.31*	*0.25*	***0.28 b***
*1/3P*	*0.86*	*0.77*	*0.87*	*0.80*	*0.29*	*0.28*	*0.28*	***0.26 ab***
*2/3P*	*0.85*	*0.79*	*0.85*	*0.84*	*0.29*	*0.27*	*0.28*	***0.23 a***
*AP*	*0.92*	*0.78*	*0.92*	*0.81*	*0.26*	*0.30*	*0.26*	***0.27 b***
Crown weight (kg)	0.10	0.14	0.10	0.15	0.19	0.19	0.18	0.18
*Pruning time*								
*2MIE*	***0.099 b***	*0.148*	***0.100 b***	*0.151*	*0.20*	*0.19*	*0.19*	*0.18*
*3MIE*	***0.095 a***	*0.141*	***0.095 a***	*0.143*	*0.17*	*0.19*	*0.17*	*0.18*
Fruit length (cm)	30.89	36.35	31.14	36.94	0.10	0.10	0.08	0.08
*Pruning time*								
*2MIE*	*30.80*	*36.44*	*31.19*	*37.04*	*0.09*	*0.09*	***0.09 b***	*0.08*
*3MIE*	*30.98*	*36.26*	*31.10*	*36.83*	*0.08*	*0.09*	***0.08 a***	*0.08*
Infructescence length (cm)	15.27	15.66	15.45	16.03	0.16	0.15	0.15	0.14
Crown length (cm)	15.63	20.70	15.70	20.91	0.11	0.14	0.11	0.13
*Pruning treatments*								
*NP*	*15.60*	*20.74*	*15.65*	*20.99*	***0.10 a***	*0.14*	*0.10*	*0.13*
*1/3P*	*15.56*	*20.53*	*15.62*	*20.84*	***0.12 b***	*0.14*	*0.12*	*0.13*
*2/3P*	*15.78*	*21.14*	*15.85*	*21.27*	***0.12 b***	*0.13*	*0.11*	*0.12*
*AP*	*15.56*	*20.35*	*15.64*	*20.52*	***0.11 ab***	*0.13*	*0.11*	*0.13*
Ratio^g^	1.06	1.36	1.05	1.34	0.21	0.22	0.21	0.21
Total soluble solids (°Brix)	13.79	15.00	13.80	15.12	0.06	0.09	0.06	0.08
Juice pH	4.04	3.81	4.05	3.84	0.03	0.05	0.04	0.05
*Pruning treatments*								
*NP*	***4.00 ab***	*3.81*	***4.01 ab***	*3.83*	*0.03*	*0.05*	*0.03*	*0.04*
*1/3P*	***4.08 bc***	*3.80*	***4.08 bc***	*3.82*	*0.03*	*0.05*	*0.03*	*0.04*
*2/3P*	***4.12 c***	*3.80*	***4.12 c***	*3.82*	*0.03*	*0.06*	*0.04*	*0.05*
*AP*	***3.96 a***	*3.85*	***3.97 a***	*3.88*	*0.04*	*0.06*	*0.04*	*0.05*
Flesh translucency	17	26	17	30	1.44	1.19	1.39	1.09

^a^: 2 months after inflorescence emergence; ^b^: 3 months after inflorescence emergence; ^c^: No slips pruned on the plants; ^d^: Slips pruned on the one-third plants with the smallest infructescences; ^e^: Slips pruned on the two-thirds plants with the smallest infructescences; ^f^: Slips pruned on all plants; ^g^: ratio crown length: infructescence length.

^h^: Values followed by the same letters in the same columns for each quality attribute, are not significantly different based on LSD (0.05). Lines in *regular font type* indicate the grand means for each quality attribute.

Individual treatment means are shown in *italic font type* for quality attributes in which the effects of pruning time, pruning treatment or their interaction were significant in one of the data sets or experiments.

Values in *bold* indicate the means in which effects were significant.

In both data sets, pruning time had no significant effect on the average fruit quality attributes, except on crown weight in Expt 1 (Table 3) where pruning at 2 MIE resulted in heavier crowns than pruning at 3 MIE (Table 4). In Expt 2, effects on crown weight were not significant.

Results also indicated that there were only small differences across experiments as shown by the grand means for the two data sets in Table 4, with slightly higher fruit and infructescence weights and lower crown weights in Expt 1 than in Expt 2, and lower fruit, infructescence and crown lengths, a lower ratio crown length: infructescence length and lower TSS in Expt 1 than in Expt 2. The juice pH was slightly higher in Expt 1 than in Expt 2 (Table 4).

### Effects of fraction of plants pruned and pruning time on the heterogeneity in fruit quality

In the data set on all plants, interaction between the pruning time and fraction of plants pruned was not significant for variation –measured as CV– in any of the quality attributes whereas main effects were only incidentally significant (Table 3). The fraction of plants pruned had only a significant effect on variation in crown length in Expt 1; fruits from plots where no slips were pruned, showed the lowest CV in crown length, although not significantly different from fruits from plots in which slips were pruned from all plants (Table 4). In Expt 2 this was not found. An effect of pruning time was only significant for variation in fruit weight in Expt 1 (Table 3) where plants pruned at 2 MIE had significantly higher CVs in fruit weight than plants pruned at 3 MIE (Table 4). This effect was not significant in Expt 2.

For the plants that had slips at pruning time, interaction between pruning time and fraction of plants pruned was significant for variation in fruit and infructescence weight in Expt 2 (Table 3); pruning of the two-thirds plants with the smallest infructescences at 3 MIE reduced significantly the CV in fruit and infructescence weight when compared with no pruning, but this was not found when pruning at 2 MIE. For variation in the other quality attributes, no main effects of the fraction of plants pruned were significant (Table 3). Comparing the two pruning times, interaction in Expt 2 indicated a significantly lower CV in fruit and infructescence weight when pruning 3 MIE compared with pruning at 2 MIE only when two-thirds of the plants were pruned. A main effect of the pruning time on the variation in other quality attributes was significant for fruit length in Expt 1 (Table 3) where pruning at 3 MIE gave lower variation in fruit length compared with pruning at 2 MIE. This was not found in Expt 2.

Results also indicated that differences in CV across experiments were in general very small, as revealed by the grand means of the CVs in Table 4. The CVs in the two experiments for the two data sets were the same for the crown weight and fruit length. For other quality attributes except the flesh translucency, differences in CVs were very small ranging from 1-3% (Table 4). For the flesh translucency, the CV was higher in Expt 1 than in Expt 2.

## Discussion

### Infructescence length and slip production

Infructescence length is an easy criterion for farmers to differentiate between plants. Our results showed that plants with higher infructescence length (in the higher sextiles) at pruning were more likely to have produced slips at pruning time (Figure 3) and produced more slips than plants with lower infructescence length (Figure 4). The higher number of slips will be related to more assimilates available in these plants and/or a better nutrient status (cf. Malézieux and Bartholomew 2003; Swete Kelly 1993). This also suggests that plants in Expt 1 –with slightly higher fruit and infructescence weight– had a better nutritional status than those in Expt 2 at the moment of pruning, what is in line with findings by Fassinou Hotegni et al. (2014b) who showed a positive association between the plant vigor at flowering induction and the slip number, fruit weight and infructescence weight at the moment of fruit harvest.

### Effects of pruning on fruit quality and variation in fruit quality

In both data sets, the fraction of plants pruned and pruning time had no consistent effects on fruit quality nor on variation in fruit quality (Tables 3 and 4). The lack of any consistent effect on average quality was surprising because slip development overlaps with fruit development and it was obvious that competition for available assimilates or nutrients within a plant might take place between the developing slips and the fruit as is the case in many crops producing fruits and side shoots, e.g. in tomato (Heuvelink 1997) and tangelo (Morales et al. 2000). Also the size of the side shoots to be removed at pruning time (Figure 2) and their number (Figure 4) were substantial. Our results agree with the results obtained with cv. Pérola in Brazil for which slip pruning did not affect any fruit variable other than crown weight (De Lima et al. 2001). In the study by De Lima et al. (2001), slips were pruned at 90 days after flowering induction; in our study, slips were pruned based on the development stage of the plant. The lack of effect of slip pruning on average fruit quality was also confirmed by Fassinou Hotegni et al. (in press) who investigated if the effect of pruning was different for plants having a different infructescence length at the moment of pruning.

The few significant effects shown by 9 out of the 240 *P*-values (Table 3) were always small (Table 4) and never consistently significant in both experiments (Table 3); they therefore most likely might have occurred by chance. The lack of an effect of pruning on quality of cv. Sugarloaf was additionally confirmed by the fact that the *P*-values in the data set containing only plants with slips were not clearly lower than the *P*-values in the data set including all plants.

Lack of effect of pruning on the average fruit quality attributes might be caused by slips becoming autotrophic at a very early stage of their development and by slips being only initiated when the plant is likely to support their growth. Kerns et al. (1936) working with Cayenne pineapple cultivar, found that during the generative phase, the infructescence is completely formed before the slips are initiated. Since the fruit is a stronger sink than other developing sinks (Malézieux et al. 2003), it would tend to take more assimilates from the plant than the other sinks. In these conditions, the slips, at the earlier stage of their development, i.e., when they appear like a bud at the upper part of the peduncle, would also take assimilates from the plants but not in a way to limit the assimilates needed for the fruit development and growth. When the slips turn from the bud stage to the leaf production stage, they certainly start producing their own assimilates for their development and growth, hence they become autotrophic. This view agrees with absence of slips or the lower number of slips produced in less vigorous plants (Figures 3 and 4); it suggests that the Sugarloaf pineapple plant adjusts the number of slips so that their need for assimilates at an early stage of development does not compromise the needs for assimilates of the fruit. The lack of a consistent significant effect of pruning on the variation in fruit quality attributes might be a direct consequence of the lack of effect of pruning on individual fruit quality.

The differences in pineapple quality between experiments (Table 4) may be related to differences in cultural practices (Table 1) and the weather conditions (Figure 1). The higher fruit and infructescence weights and smaller crown weight and lengths in Expt 1 could be related to a later moment of flowering induction (Fassinou Hotegni et al. 2014b). The lower TSS and higher pH in Exp. 1 could be related to the cooler temperatures in the last month before harvest (Paull and Chen 2003).

### Implications

Pruning of slips, either in selected plants or across all plants, did not lead to a consistent significant improvement in the average quality of the harvested pineapple fruits nor in the variation in quality compared with no pruning. Practical implications of the results are that it is not recommended to farmers to prune slips. Further studies should be done to determine how the Sugarloaf pineapple plant adjusts the available assimilates at flowering induction to the number of the side shoots to be produced.

## Abbreviations

CV: Coefficient of variation
MIE: Months after inflorescence emergence
TSS: Total soluble solids
